# David vs. Goliath: The Structure, Function, and Clinical Prospects of Antibody Fragments

**DOI:** 10.3390/antib8020028

**Published:** 2019-04-09

**Authors:** Adam Bates, Christine A. Power

**Affiliations:** Biopharm Molecular Discovery, GlaxoSmithKline, Hertfordshire SG1 2NY, UK; adam.x.bates@gsk.com

**Keywords:** ADC, antibody fragments, BiTE^®^, diabodies, domain antibodies, fab, ImmTAC^®^, Nanobody^®^, scFv, TandAb, V-NAR

## Abstract

Since the licensing of the first monoclonal antibody therapy in 1986, monoclonal antibodies have become the largest class of biopharmaceuticals with over 80 antibodies currently approved for a variety of disease indications. The development of smaller, antigen binding antibody fragments, derived from conventional antibodies or produced recombinantly, has been growing at a fast pace. Antibody fragments can be used on their own or linked to other molecules to generate numerous possibilities for bispecific, multi-specific, multimeric, or multifunctional molecules, and to achieve a variety of biological effects. They offer several advantages over full-length monoclonal antibodies, particularly a lower cost of goods, and because of their small size they can penetrate tissues, access challenging epitopes, and have potentially reduced immunogenicity. In this review, we will discuss the structure, production, and mechanism of action of EMA/FDA-approved fragments and of those in clinical and pre-clinical development. We will also discuss current topics of interest surrounding the potential use of antibody fragments for intracellular targeting and blood–brain barrier (BBB) penetration.

## 1. Introduction

Since the licensing of the first monoclonal antibody (mAb) therapy, Orthoclone^TM^ (OKT3), in 1986, the specificity, flexibility, and diversity of antibodies and antibody derivatives has led to their becoming the largest class of biopharmaceuticals [1]. Monoclonal antibodies have a history of safe use, a strong scientific basis, and a high degree of technical feasibility. As of October 2018, over 80 antibodies were marketed or approved by the EMA/FDA for multiple disease indications including cancer, inflammation/autoimmunity, transplantation, infectious and cardiovascular diseases, haematology, allergy, and ophthalmology. Over 500 more, including 2nd generation products and novel antibody formats, are currently in clinical trials around the world [1,2,3].

The modular nature of antibodies, both structurally and functionally, allows for the generation of smaller antigen binding fragments, such as fragment antigen binding (Fab), the single chain fragment variable (scFv), single-domain antibodies, and the fragment crystallizable (Fc) domain, through molecular cloning, antibody engineering, and even enzymatic methods. Antibody fragments can then be used on their own or linked to other molecules or fragments to generate bispecific, multi-specific, multimeric, or multifunctional molecules to achieve a variety of biological effects [4]. 

Antibody fragments can offer several advantages over the use of conventional antibodies. For example, they can be produced easily, generally using microbial expression systems, which results in faster cultivation, higher yields, and lower production costs [5]. Their small size allows access to challenging, cryptic epitopes, and tumour penetration, they have reduced immunogenicity, and the lack of Fc limits bystander activation of the immune system [6]. On the other hand, their smaller size results in faster renal excretion, which may require higher doses and/or more frequent dosing regimens in vivo unless mitigated by the addition of half-life extension moieties such as polyethylene glycol or albumin binding fragments. 

In this review, we describe the history, structure, formats, mechanisms of action, and production of some of the most common antibody fragments. We also discuss some of the formats currently being tested in clinical and non-clinical settings, as well as briefly touching on future applications of this expanding class of biopharmaceuticals. 

## 2. Antibody Fragment Formats

### 2.1. Fragment Variable (Fv)-Based Formats

#### 2.1.1. Single Chain Fragment Variable (scFv)

**Figure 1:**
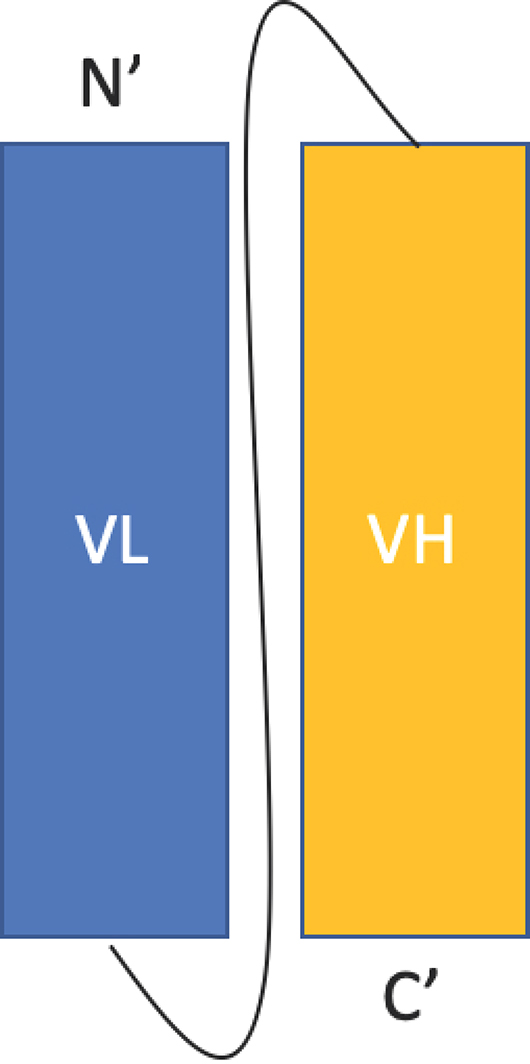
The single chain fragment variable format. The C-terminus of the light chain (VL) is linked the N-terminus of the heavy chain (VH) by a flexible glycine- and serine- rich linker.

The single chain fragment variable (scFv) was first described in 1988 by Bird et al. [7] and comprises the variable regions of the light chain (VL) and heavy chain (VH) of an antibody linked by a flexible peptide, which is most commonly glycine- and serine-rich with dispersed hydrophilic residues (Figure 1), to produce a single chain protein with an affinity for its antigen comparable to that of the parental mAb. The sequence and length of the ideal linker may differ between scFvs in order to optimise affinity for the antigen and thermostability. It is believed that the linker must span a ~3.5 nm distance between the VL and VH without disrupting the formation of the antigen binding site [8].

The scFv has several advantages over a conventional mAb. Firstly, their small size of approximately 27 kDa makes them ideal for large-scale production in microbial systems [9]. Additionally, as the VL and VH coding sequences are genetically linked in a single transcript, there is no need to balance the expression of the light chain (LC) and heavy chain (HC). This allows fragments to be produced more quickly, in higher yields, and at lower costs than full-sized mAbs, which generally require mammalian expression systems [10]. Their small size also facilitates tissue penetration and access to cryptic epitopes, making them especially useful for tumour penetration in cancer immunotherapy [11,12]. The lack of an Fc region removes the risk of bystander immune cell activation and antibody effector functions such as antibody-dependent cellular cytotoxicity (ADCC), antibody-dependent cellular phagocytosis (ADCP), or complement-dependent cytotoxicity (CDC), allowing the molecule to bind its target without activation of the host’s immune system [6]. This may be advantageous or disadvantageous depending on the context.

The lack of an Fc domain also brings about several disadvantages, mainly low thermostability compared to the parental mAb, a greater propensity for aggregation, therefore increasing the risk of immunogenicity, and a shorter half-life due to a lack of FcRn-mediated recycling. This can lead to the need for higher and more frequent dosing [4,13,14]. Fusion of the scFv to albumin or polyethylene glycol (PEG) can be used to improve half-life. However, such fusions can offset the advantages that an scFv holds over a mAb due to cost and the increase in size [15,16]. Examples of scFvs in clinical development include gancotamab (Merrimack Pharma), pexelizumab (Alexion), and Novartis’s brolucizumab [17].

#### 2.1.2. Tandem scFvs

**Figure 2:**
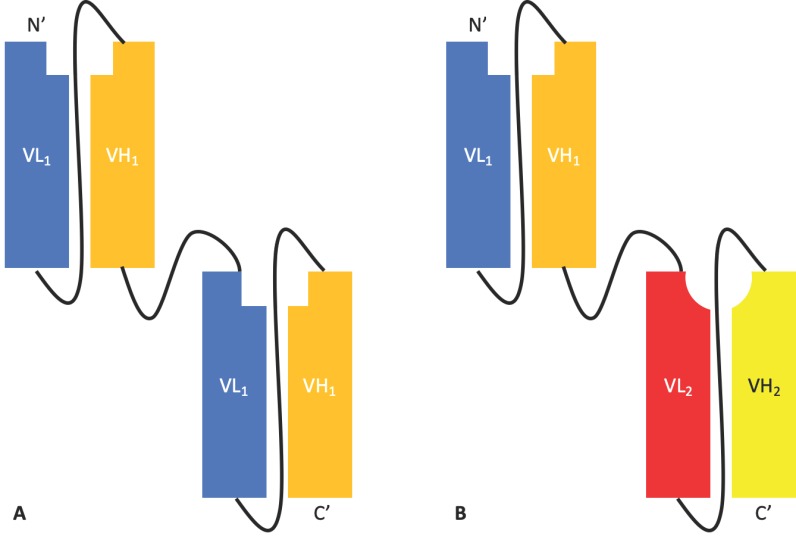
The tandem scFv platform. (**A**) A monospecific bivalent tandem scFv composed of two identical scFvs joined by a helical linker. (**B**) A bispecific bivalent scFv composed of two different scFvs joined by a helical linker.

Tandem scFvs such as Micromet AG’s solitomab [17] are an adaptation of the scFv format. When two different scFvs are linked, the tandem scFv is arguably the simplest bispecific antibody platform [18]. A tandem scFv links two or more scFvs through helical peptide linkers in the orientation NH_2_–VL_1_–VH_1_–(linker–VL_2_–VH_2_)_n_–COOH, resulting in a single chain bivalent and bi-specific molecule encoded by a single gene [19] (Figure 2). These can be used to target one antigen with increased avidity, to target two distinct antigens simultaneously, or even to target albumin, thus increasing half-life [20].

#### 2.1.3. Diabodies, DART^®^s, and TandAbs

**Figure 3:**
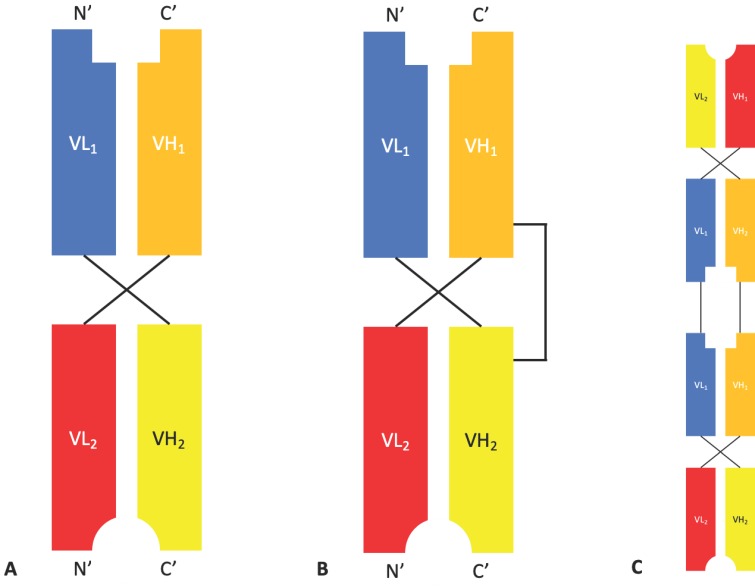
The structures of diabody, DART^®^, and TandAb fragments. (**A**) A bispecific diabody composed of two different chains, each containing a VL and VH from different antibodies, in a head-to-tail arrangement. (**B**) A bispecific dual affinity re-targeting (DART^®^) protein containing two distinct polypeptide chains held together by non-covalent interactions and a disulphide bond. (**C**) A TandAb composed of two diabodies linked in a linear arrangement to produce a tetravalent bispecific molecule.

Diabodies are bivalent dimers formed from two chains, each containing a VH and a VL domain. The two domains within a chain are separated by a pentameric glycine-rich linker (G_4_S) that is too short to facilitate intrachain dimerization leading to two chains dimerising in a head-to-tail arrangement. By using two different chains with the same orientation, the first containing the VH of Antibody 1 and the VL of Antibody 2, and the second containing the VH of Antibody 2 and the VL of Antibody 1, bispecific bivalent dimers are produced [21] (Figure 3). A study comparing different formats of bispecific diabodies showed that not all possible diabody formats retain binding to both antigens, highlighting the importance of domain arrangement and orientation [22]. 

Over time, additional modifications have been made to the diabody format to further improve stability. Dual affinity re-targeting proteins (DART^®^s) (Figure 3) developed by MacroGenics, contain an interdomain disulphide bond for increased stability that results in a structure that is rigid and compact [23]. MacroGenics currently have four DART^®^s in phase I clinical trials for oncology, autoimmune disease, and HIV infection [24]. 

Tri- and tetra-valent molecules with a structure similar to that of a diabody can be produced by linking three or four variable domains together in a single chain. Affimed specialise in the development of TandAbs, tetravalent bispecific molecules composed of two diabodies fused in a linear fashion (Figure 3), through their proprietary ROCK^®^ platform. There are currently five ongoing clinical trials involving a selection of T-cell and natural killer (NK) cell engagers for the treatment Hodgkin’s lymphoma and acute lymphocytic leukaemia (ALL) [25].

A major advantage of diabodies and tandem scFvs is their bivalency and ability to bring two targets into proximity. Furthermore, diabodies and tandem scFvs are bispecific-compatible formats, which has made them promising molecular formats for cancer immunotherapy, e.g., bispecific T-cell engagers (BiTE^®^s) [26,27].

#### 2.1.4. Bispecific Fv Fusion Antibodies with an Fc Domain

**Figure 4:**
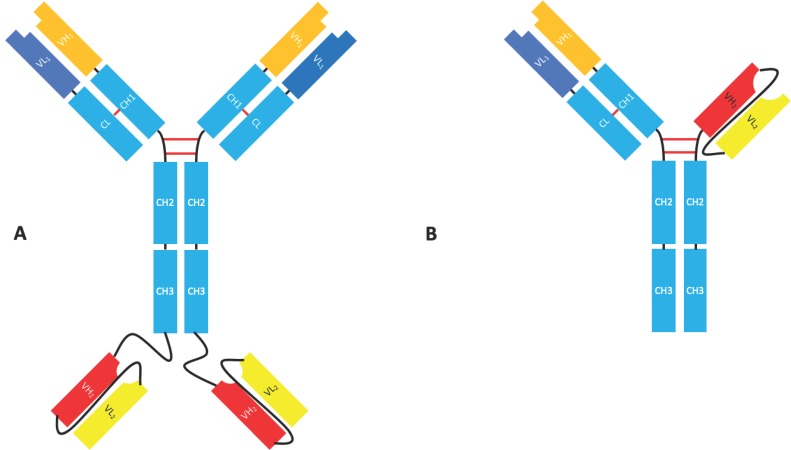
scFv fusion bispecific formats with an Fc domain. (**A**) IgG-scFv. Canonical IgG with a scFv fused to the C-terminus of the CH3 domain to produce a tetravalent bispecific molecule. (**B**) Fab-scFv-Fc IgG with one IgG Fab arm exchanged for a scFv.

Although not fragments in their own right, there are many bispecific formats such as the IgG-scFv, which uses an scFv to target an additional epitope or antigen (Figure 4). As well as being a flexible format that allows for the production of multivalent multi-specific molecules with a modifiable effector function, these molecules can easily be produced recombinantly [18,28,29]. Istiratumab, an IgG-scFv developed by Merrimack Pharmaceuticals, is currently in clinical trials for hepatocellular carcinoma and pancreatic cancer [17]. Fab-scFv-Fc (Figure 4) is another bispecific format that uses an scFv to grant specificity for a second epitope. The format is being used in molecules such as Zymeworks’ ZW25, an anti-Her2/Her2 bispecific (biparatopic) antibody in phase I clinical trials for the treatment of Her2 expressing cancers. Xencor have four bispecific molecules with this format in phase I clinical trials for the treatment of acute myeloid leukaemia (AML), B-cell tumours, and neuroendocrine tumours [30].

### 2.2. Fab Based Formats

#### Fab & F(ab’)_2_ Formats

**Figure 5:**
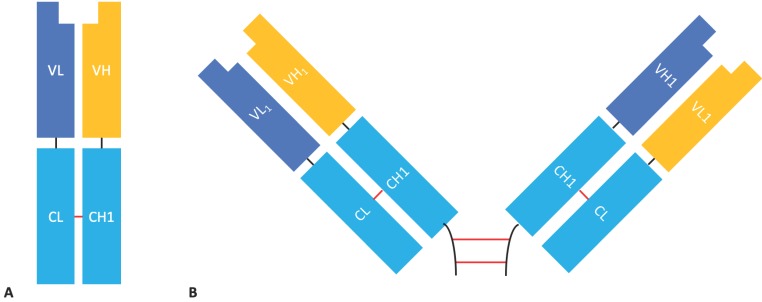
The structure of Fab and F(ab’)_2_ fragments. (**A**) Fab fragment composed of an LC (containing VL and CL) linked to an Fd (containing VH and CH1) by a disulphide bond between the CL and CH1 domains. (**B**) F(ab’)_2_ fragment composed of two Fab fragments joined by an IgG hinge region.

The fragment of antigen binding (Fab) (Figure 5) was the first therapeutic antibody fragment format and remains one of the most successful, laying claim to eight molecules entering clinical trials pre-1995 and comprising ~49% of all antibody fragments that have entered clinical trials [4,31]. There are currently three FDA-approved Fabs: abciximab (Reopro^®^), idarucizumab (Praxbind^®^), and ranibizumab (Leucentis^®^) [17].

Fab fragments are composed of an antibody light chain (VL + CL domains) linked by a disulphide bond to the antibody heavy chain VH and CH1 domains; the molecular format is monovalent and monospecific and retains the parental antibody’s ability to bind its antigen with high specificity and affinity. Fabs share many of the characteristics of scFvs. Although Fabs are not as small, and therefore presumably not as good at penetrating tissue as scFvs [12], with a mass of ~50 kDa they are much smaller than mAbs (the MW of IgG is ~150 kDa). Fabs lack an Fc domain, reducing the risk of immune cell bystander activation and non-specific binding [6] and allowing for easier production at the expense of increased aggregation, lower stability, and reduced half-life [5,32]. Fab fragments are more stable than their scFv counterparts due to the mutual stabilisation that occurs between the VH/VL and CH1/CL interfaces [33]. They also have the advantage of being completely native structures and as such they avoid the time and resources required to engineer an ideal linker and are therefore less likely to be immunogenic. 

F(ab’)_2_ fragments are composed of two Fab fragments held together by an Ig hinge region and have a molecular mass of ~110 kDa (Figure 5). F(ab’)_2_ fragments are bivalent, giving them increased avidity compared to Fabs [32], but their larger size may lead to reduced tissue penetration. However, their tissue penetration is still superior to that of a full-sized mAb [12]. They can be generated by the enzymatic digestion of full-length antibodies or by the expression of the recombinant F(ab’)_2_ in mammalian cells.

Like scFvs, Fab fragments suffer from a reduced half-life compared to their parental mAbs due to the lack of an Fc domain. This removes the possibility of FcRn-mediated recycling leading to rapid degradation of the Fab-antigen complex after absorption by macropinocytosis [34,35,36]. The half-life of Fab fragments can be extended in a similar manner to the half-life extension of scFvs, typically by conjugation to PEG or fusion to an albumin binding protein [16,37,38]. These Fab conjugate proteins have had some success with multiple molecules in clinical trials or having already received FDA approval such as certolizumab pegol (Cimzia^®^), a marketed PEGylated anti-TNFα Fab for rheumatoid arthritis.

### 2.3. Single-Domain Antibodies

#### 2.3.1. Nanobodies

**Figure 6:**
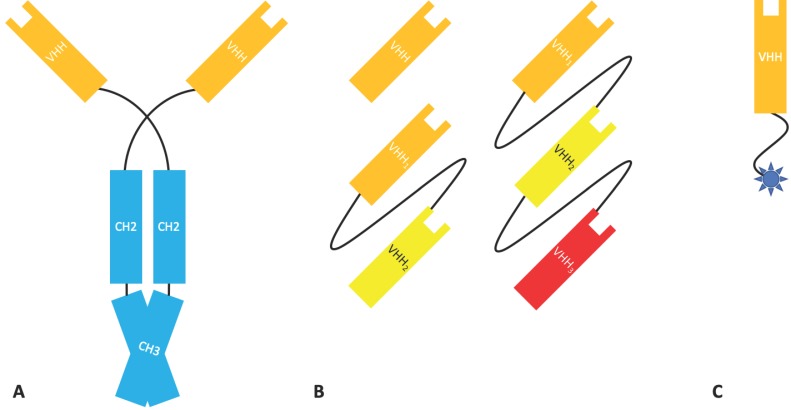
Camelid heavy-chain IgG and Nanobody^®^ fragments. (**A**) The structure of heavy-chain IgG, composed of two heavy chains, each containing a VHH domain, a CH2 domain, and a CH3 domain. (**B**) Mono-, bi-, and tri-valent Nb formats with each VHH having a different antigen specificity. (**C**) A Nanobody^®^ drug conjugate.

Nanobodies^®^ (Nbs), a class of antibody fragment developed by Ablynx, are recombinantly expressed antigen binding VHH domains from heavy-chain IgG, a type of immunoglobulin found in camelids [39]. With an MW of 12–15 kDa, Nbs are one of the smallest naturally occurring antigen binding fragments. Heavy-chain IgG (Figure 6) is devoid of light chain, lacks the CH1 domain found in mammalian immunoglobulins, and therefore binds its antigen bivalently solely through two VHH domains. Isolated VHH domains retain the ability to bind their antigen and are robust under stringent conditions. Nbs can resist a wide pH range and high temperatures and have been shown to tolerate the presence of organic solvents (although these characteristics are not present in all Nbs). They are also highly soluble and, due to their small size and extended CDRH3 loop, can rapidly penetrate tissue and access cryptic epitopes [40,41,42]. Such molecules are also easy to produce recombinantly without issues relating to inter-domain interactions, such as those found in scFvs and Fab fragments [43]. Although not of human origin and frequently ‘humanised’, Nbs are rarely immunogenic due to their small size and similarities with the human VH3 gene family [43]. 

Fusion of Nbs that bind different epitopes allows the creation of multivalent molecules with high affinity or potency. The monomeric behaviour, good solubility, and modular nature of Nbs also allow them to be easily fused to other Nbs with different antigen specificities or covalently linked to other molecules [44].

Unfortunately, the small size of Nbs is below renal cut-off, leading to rapid renal clearance with a half-life of approximately 2 h, making them particularly unsuitable for chronic use in many therapeutic areas [43,45]. However, the fast clearance is a beneficial property for in vivo diagnostics (see Section 5.1). Strategies to extend their half-life, including PEGylation, conjugation to the Fc domain of conventional antibodies, and coupling to abundant serum proteins such as human serum albumin (HSA), apolipoprotein L1, and β-Lactamase, have been explored. Fusion of Nbs to an anti-albumin VHH has been validated in the clinic [43]. Fusion of a VHH to the Fc of IgG has also been investigated as a means to produce bispecific tetravalent molecules [46].

The favourable characteristics of VHH domains has led to excitement over their potential. With 5 Nbs currently in clinical trials, and the recent approval of caplacizumab (Cablivi^TM^), a bivalent VHH targeting von Willebrand factor (vWF), by the EMA in October 2018 and the FDA in February 2019 for thrombotic thrombocytopenic purpura and thrombosis, this excitement is not surprising [17].

**Figure 7:**
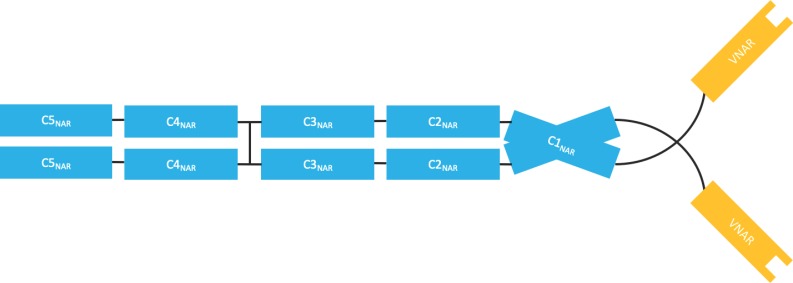
Shark heavy chain antibody (Ig-NAR), a dimer of heavy chains containing five constant domains and the antigen binding variable nucleotide antigen receptor (V-NAR).

Analogous to the camelid nanobodies is the single variable new antigen receptor (V-NAR), domain antibody fragments obtained from cartilaginous fishes such as sharks (Figure 7) [47]. Whilst V-NARs share some similarities in terms of their structure and properties with domain antibodies, they are smaller (11 kDa) and more stable proteins. Contrary to mammalian variable domains, V-NAR domains have only two complementarity determining regions, CDR1 and CDR3. However, they also contain two mutation-prone regions named HV2 and HV4; the latter has been shown to contribute to antigen binding [48,49]. As V-NARs are evolutionarily derived from a non-antibody lineage, this arguably places them outside the complex and competitive antibody patent landscape. The V-NAR format is being developed by Elasmogen (UK) under the name of soloMERs^TM^.

#### 2.3.2. Domain Antibodies

**Figure 8:**
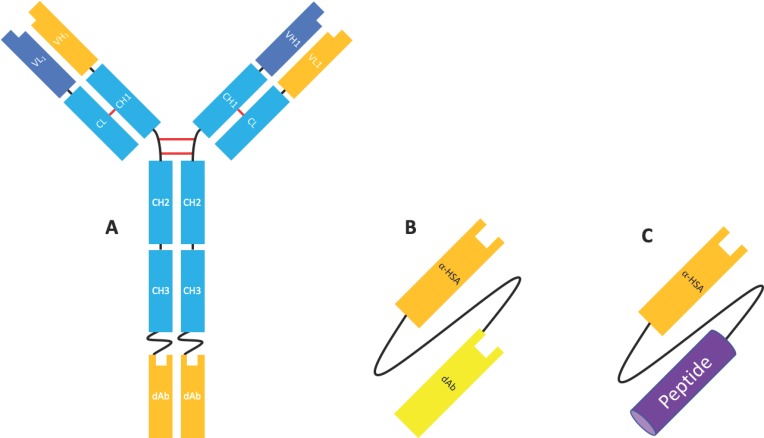
The uses of domain antibodies (dAbs). (**A**) IgG-dAb (also called a mAb-dAb). IgG with a dAb fused to the C terminus of each heavy chain to produce a bispecific tetravalent molecule. (**B**) Tandem dAb with an anti-HSA domain. The dAb against the target of interest is linked to an anti-HSA dAb to improve half-life. (**C**) AlbuDab^®^. A peptide linked to an anti-HSA dAb to improve the peptide’s half-life.

Domain antibodies (dAbs) are fully human unpaired variable domains (either VH or VL), which have been engineered to prevent dimerization whilst maintaining the specificity and affinity of a canonical antigen binding site (VH and VL). This engineering commonly involves ‘camelisation’, in which the hydrophobic residues usually found at the VH/VL interface are substituted for hydrophilic residues found in camelid VHH and in the extension of the CDRH3 [50,51]. Similar in size and structure, these molecules possess many of the advantageous properties of Nbs, including high thermostability, high solubility, and a short half-life and are amenable to conjugation/fusion and high-yield microbial expression [51]. Although naked dAbs may have some application as therapeutic molecules (see Section 4.1 and Section 4.2), dAbs have been most extensively investigated as fusion proteins to other moieties, such as full-length antibodies to enable specificity for a second antigen [18,52], an Fc domain (e.g., Placulumab), or with an anti-albumin dAb (e.g., GSK/Domantis’ AlbudAb^®^s) [53] (Figure 8).

Albudab^®^s (Figure 8) contain an anti-HSA binding domain which can increase the half-life of the species to 19 days, the half-life of HSA. GSK2374697 was an AlbudAb^®^ developed for type 2 diabetes which phase I clinical trials were reached. It was composed of exendin-4, a GLP-1 mimetic peptide isolated from Gila monster saliva, linked to an anti-HSA dAb as a single transcript. The anti-HSA dAb extended the half-life of the peptide from 30 min to 6–10 days [53].

## 3. The Production of Antibody Fragments

### 3.1. Expression

Conventional full-length mAbs contain an N-linked glycosylation site in the CH2 domain of the heavy chain which is important for stability, preventing aggregation, and effector function [54]. Aberrant glycosylation can cause unfavourable molecular properties and be highly immunogenic. As such, the ability to produce post-translational modifications that closely resemble those that occur naturally necessitates expression in mammalian cell lines such as Chinese hamster ovary (CHO) cells, human embryonic kidney (HEK) cells, and to a lesser extent NS0 murine myeloma cells and human PER.C6^TM^ cells [55].

Unlike full-length mAbs, antibody fragments are not generally glycosylated. Fragments are therefore amenable to production in microbial systems, allowing for faster, cheaper production in cell lines that are easier to cultivate and manipulate [10].

#### 3.1.1. *Escherichia coli*

*Escherichia coli* was the first microbial system used for the production of biopharmaceuticals and is still used for the production of ~40% of biopharmaceuticals available today, including certolizumab pegol and ranibizumab [10,17]. As a prokaryote, *E. coli*’s rapid growth in inexpensive media, well understood genetics, and high manipulability make it an ideal expression system when glycosylation is not required.

Traditionally, the gene(s) of interest was placed on a self-replicating, high copy number plasmid under the control of a promoter such as the bacteriophage T7, lac operon (*lac*), or tryptophan (*trp*) promoter alongside a selectable marker. However, the greater volumetric productivity that can be achieved using this method is offset by the inhibition of cell growth and cell death caused by the metabolic burden of antibody fragment production. Recently, marker-free and plasmid-free expression systems have been developed to reduce this metabolic burden and allow for greater overall yields [10,56,57].

There are two main established approaches for the production of antibody fragments in *E. coli*, the first of which is to express the protein of interest in *E. coli*’s reducing cytoplasm. This method allows for high yields of protein, but the reducing conditions are not permissive for disulphide bond formation, and inclusion bodies are regularly formed. The protein must then be re-folded after purification which can be time-consuming, inefficient, and costly [10]. This issue can be somewhat alleviated by the co-expression of chaperones to facilitate correct protein folding [58]. The second pathway involves targeting, by fusion to an N-terminal leader peptide such as Pel B, the protein of interest to the oxidising periplasm, where disulphide bonds can readily form [59,60]. This second method does result in lower yields; therefore, if efficient re-folding of the antibody fragment is possible, cytoplasmic expression may be preferable.

Finally, due to the ability of *E. coli* to grow at high cell densities, antibody fragments are commonly produced in high cell density cultures grown in a stirred tank reactor using a fed-batch method [61].

#### 3.1.2. *Saccharomyces cerevisiae*

*Saccharomyces cerevisiae* was the first yeast used as an expression system for recombinant proteins. Its high genomic stability, manipulability, and ease of cultivation have made it a strong choice for the expression of antibody fragments. Additionally, *S. cerevisiae* is used in a well-established production method of llama VHH fragments, consistently giving yields of hundreds of milligrams per litre [62].

There are three commonly used vector systems with *S. cerevisiae*. Firstly, a yeast episomal plasmid containing an origin of replication allows for gene expression with a high plasmid copy number without genomic integration [10]. Secondly, yeast centromeric plasmids containing a self-replicating sequence allow for gene expression with a single or low plasmid copy number without genomic integration [10]. Finally, yeast integrative plasmids do not contain an origin of replication but rather are incorporated into the yeast’s genome, leading to improved process quality and stability at the cost of expression levels [10,63]. However, methods have been developed to circumvent this issue such as targeted integration of the gene of interest at the ribosomal DNA locus, a highly-transcribed region [64]. Glyceraldehyde-3-phosphate dehydrogenase (GAPDH), alcohol dehydrogenase 1 (ADH1), and phosphoglycerate kinase 1 (PGK1) are promotors from *S. cerevisiae’s* native glycolytic pathway that are commonly used to achieve high expression levels, and methods have been developed to allow the co-expression of multiple genes on self-replicating plasmids making *S. cerevisiae* suitable for the expression of multi-gene fragments such as Fabs [65,66].

A major issue that plagues *S. cerevisiae* is endoplasmic reticulum (ER) misfolding and inefficient trafficking of the protein of interest leading to the accumulation of misfolded protein in the ER or vacuolar-like structures [10]. Although *S. cerevisiae* has proven to be an excellent expression system for VHH domains [41], this issue is particularly apparent with the more hydrophobic scFvs [67]; however, simultaneous overexpression of chaperones and foldases has been shown to facilitate scFv secretion [68].

Similar to *E. coli*, *S. cerevisiae* is usually grown in glucose-limited fed-batch culture [62]. The limited glucose helps prevent the depletion of oxygen and switch to fermentative metabolism, which leads to the undesirable production of toxic metabolites [69].

#### 3.1.3. *Pichia pastoris*

*Pichia pastoris* can be used as an alternative to *S. cerevisiae* and uses an integrated vector to achieve stable expression of the protein of interest.

*P. pastoris* metabolises methanol as its sole source of carbon and, as such, expresses large amounts of alcohol oxidase. The alcohol oxidase promoter was commonly used to express proteins of interest, but expression was hard to control. More adjustable promotors are now being investigated [70].

The genome of several strains of *P. pastoris* have been published online along with a genome scale metabolic model allowing for straightforward engineering and strain optimisation [71].

The preference of *P. pastoris* for respiratory over fermentative growth allows it to be grown to much higher cell densities than *S. cerevisiae* using inexpensive media, usually using a fed-batch method [72]. A *P. pastoris* system has been used to produce ALX-0171, a trimeric nanobody being developed for RSV infection [73], scFv-h3D6, an anti Aβ antibody fragment which is being developed for Alzheimer’s disease [9], and scFvTEG4-2c against platelet anti-αIIbβ3, for potential use as an imaging agent for atherosclerosis [74].

#### 3.1.4. Cell-Free Expression Systems

There has recently been significant interest in cell-free production of antibody fragments. Approaches using *E. coli* cell lysates [75], CHO cell lysates [76], and insect cell lysates [77] have been successfully used to produce antibody fragments. Cell-free expression systems have been shown to be fast, reliable, flexible, and scalable [78]. Of note is the ability todirectly input linear DNA encoding the protein of interest rather than constructing a complex plasmid for transformation and the selection of transformed cells. Additionally, the lack of a phospholipid bilayer barrier allows forthe simple addition of resources required for the production of polypeptides, such as amino acids and ATP, and supplements, such as chaperones, the prokaryotic disulphide bond isomerase disulphide bond c (Dsbc), and oxidised/reduced glutathione rather than the co-expression or simultaneous over-expression required with cell-based systems. 

Although not usually suitable for the commercial scale production of recombinant proteins, cell-free systems have been successfully used to produce functional scFvs [79] and more recently, using both prokaryotic and eukaryotic systems, functional Fabs [78,80]. Pioneered by companies like Sutro Biopharma, cell-free systems have been used to produce scFvs conjugated to moieties, such as granulocyte-monocyte colony stimulating factor (GM-CSF) and interleukin 1β-derived peptide [81] in a scalable manner, and can be used for the incorporation of non-canonical amino acids for site-directed conjugation, making cell-free expression an optimal system for protein engineering [82]. Such non-canonical amino acids are often toxic to cells or are unable to cross the cell membrane, making them difficult to incorporate whilst using cell-based systems. Although not currently a preferred method, the rise of antibody drug conjugates and improvements to cell-free expression systems may soon increase the desirability of cell-free expression systems for antibody fragment production. Sutro’s cell-free antibody production system was used to make STRO-001, a novel CD74 targeting antibody drug conjugate in phase I clinical trials for B-cell malignancies including multiple myeloma and non-Hodgkin’s lymphoma [83].

### 3.2. Enzymatic Cleavage

Although recombinant expression is the method most commonly used for the production of antibody fragments, fragments such as Fab, F(ab’)_2_, and Fc can be easily produced from their parent mAb by enzymatic cleavage using commercially available enzymes. Enzymatic cleavage can be preferable to recombinant expression for the production of certain fragment formats, such as F(ab’)_2_, which has a propensity to aggregate when expressed recombinantly due to the hinge region. This was originally done using papain, which cleaves just above of the hinge region to produce two Fab fragments and a hinge-CH2-CH3 fragment [84], or pepsin, which cleaves just below the hinge region to produce an F(ab’)_2_ and an Fc fragment [85]. Companies like Genovis now produce improved versions of these enzymes, allowing for a more efficient production of antibody fragments from the parental mAb [86].

### 3.3. Purification

The purification of antibody fragments is somewhat more complicated than the purification of full-sized mAbs, owing to the lack of an Fc domain which facilitates efficient purification by Protein A or Protein G affinity chromatography [87,88]. However, VH 3 family containing fragments can be purified using Protein A [89]. Currently, there are no ‘toolbox’ or generic approaches to the production of pharmaceutical antibody fragments, with the current approved fragment therapies being purified using different combinations of chromatographic and non-chromatographic techniques [90]. In theory, an antibody fragment could be captured selectively using its antigen fixed to a resin. Whilst this may be used in some cases, antigens are not always readily available and prior production, purification, and fixation of said antigen would have a significant impact on the cost of goods.

The unique nature of fragments and the lack of large conserved regions pose an additional challenge to the development of generic purification approaches. To this end, micro-fluidic approaches have been tested to quickly determine the ideal binding conditions of specific fragments to inform future purification attempts [91]. 

If the expression system used expresses the fragment in the cytoplasm or periplasm rather than secreting it, the fragments are first freed by lysing the cells, and proteins are then refolded if necessary. Once this has been completed, the fragment can be purified using one or more of the methods described in the following sections.

**Figure 9:**
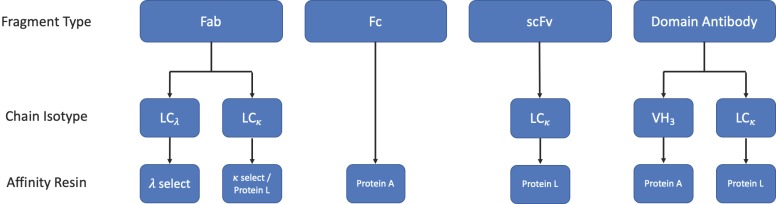
Simplified purification workflow for different fragment formats. A series of stages that can be used to purify common antibody fragments based on the presence of certain domains. The workflow typically includes affinity chromatography (where applicable) followed by multi-modal polishing stages, which may include size exclusion chromatography (SEC), cation (CIEX) or anion (AIEX) exchange, and hydrophobic interaction chromatography (HIC). Antibody fragments can also be purified using cation exchange chromatography (CIEX) or immobilised metal affinity chromatography (IMAC) as the initial capture step.

A simplified workflow for the purification of applicable fragments by affinity chromatography is shown in Figure 9.

#### 3.3.1. Protein L Affinity Chromatography

Protein L is a cell wall-associated protein isolated from *Peptostreptococcus magnus* [92], which binds strongly to human Ig LCs, scFv, and Fab fragments [93]. Protein L targets a site that lies within the variable region of κ 1, 3, or 4 light chains, allowing it to capture a wide range of mAbs and fragment formats. However, should the fragment of interest be derived from a λ mAb or from the κ 2 sub-family, protein L would be unable to capture the fragment [93]. In cases such as these, commercially available κ- and λ-select resins may be suitable alternatives.

#### 3.3.2. Affinity Tags

As antibody fragments are most commonly produced recombinantly, they can easily be generated with affinity tags such as hexa-histidine (6HIS), glutathione-S transferase (GST), or mannose binding protein (MBP) using a cleavable linker to allow purification by immobilised metal affinity chromatography (IMAC) or other affinity-based methods [94]. Such techniques would allow for the selective capture of the desired fragment regardless of the Ig germline family. However, affinity tags are not a blanket solution. Removal of the affinity tag by proteolytic cleavage may leave residual amino acids, which may cause issues with aggregation, misfolding, and immunogenicity. It is also important to consider the host’s cellular proteases when designing the linker to avoid premature cleavage and production of irrecoverable material [95,96]. Additionally, a study carried out by Das et al. showed that Protein L affinity chromatography, where applicable, was a more robust and versatile method for the purification of scFvs than IMAC using a (6HIS) tag [97].

#### 3.3.3. Other Chromatographic Methods

As well as the affinity-based methods described above, other chromatographic methods such as size exclusion chromatography, ion exchange chromatography, and multi-modal chromatography are used to separate the desired fragment from contaminants based on size and isoelectric point [98,99]. Ion exchange chromatography usually takes the form of cation-exchange chromatography (CIEX) and has the added advantage of being able to separate charge variants [100]. In order to achieve the high purity required for pharmaceutical applications, these chromatographic methods are used in conjunction with each other, with affinity-based methods, and/or with non-chromatographic methods [90]. More recently, multi-modal approaches have been used in the purification of Fab fragments and have been shown to be superior to traditional CIEX resins due to increased salt tolerance and Fab binding [91,101].

## 4. Antibody Fragments in the Clinic

### 4.1. Oncology

The majority of antibody fragments currently being developed in the clinic are for oncological applications. In addition to the generic characteristics of antibody fragments that make them attractive as immunotherapies, e.g., their small size, which grants them superior tissue and tumour penetration compared to a conventional mAb [12], and the lack of an Fc domain that reduces non-specific activation of innate immune cells, there are many mechanisms of action that are unique to a specific format. The diversity of formats being investigated for their therapeutic potential in oncology is astounding, but the majority of fragments are reformatted as bispecific molecules combining an anti-CD3 binding moiety with an anti-tumour binding domain. For more detailed information, the reader is referred to two recent excellent reviews by Wu et al. and Velasquez et al. [102,103]. Here we will only cover the more established formats and describe the over-arching pathways that they exploit.

#### 4.1.1. BiTE^®^s

**Figure 10:**
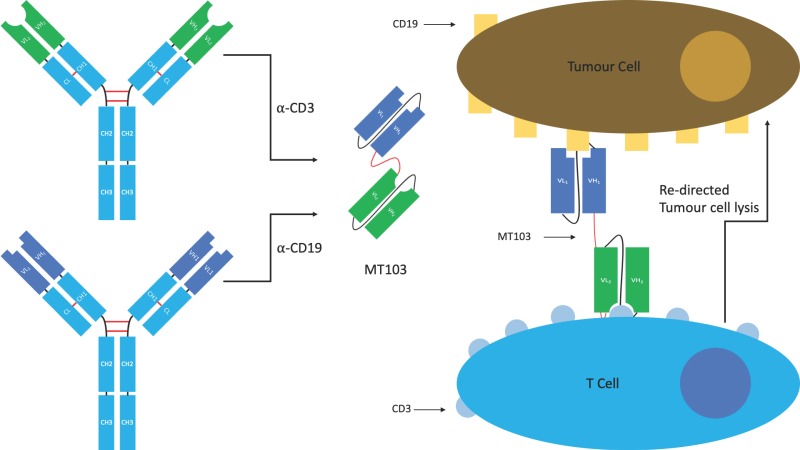
The structure and mechanism of action of MT103, an α-CD3/α-CD19 bispecific T-cell engager (BiTE^®^). The antigen binding site of each parental antibody is isolated and converted into an scFv format. The two scFvs are then joined by a flexible peptide linker to produce a bispecific moiety. The anti-CD19 scFv binds to tumour cells whilst the anti-CD3 scFv will bind passing T-cells, re-directing them to attack the tumour cell.

Bispecific T-cell engagers (BiTE^®^s) originally developed by Micromet, are a specific class of tandem scFv used to redirect cytotoxic T-cells to tumours. The induced response is highly selective for the target tumour cells, more so than can be achieved by radio- or chemotherapy. The hope is that this selectivity will lead to reduced off-target effects (Figure 10) [26]. 

BiTE^®^s contain two antigen binding sites. The first is directed against a tumour antigen, whilst the second is directed against the T-cell receptor (TCR) signalling complex CD3. Simultaneous binding of the BiTE^®^ to CD3 and the tumour antigen (e.g., CD19) bypasses pMHC restriction and induces T-cell activation, cytokine production, the formation of cytolytic immunological synapses leading to a tumour-directed cytotoxic response, and the activation of other host immune responses [26,104]. The use of a monovalent anti-CD3 is thought to be important in limiting off-target immune activating functions that can lead to cytokine release syndrome and cytokine storm, a problem seen with some of the early anti-CD3 mAbs in the clinic such as muromonab (OKT3), which eventually led to its withdrawal.

There is currently one licenced BiTE^®^ which received FDA approval in 2017. Blinatumomab (Blincyto^®^), developed by Amgen and Astellas Pharma Inc., is an anti-CD19/CD3 BiTE^®^, used for the treatment of Non-Hodgkin’s lymphoma and Philadelphia chromosome negative acute lymphoblastic leukaemia. It is also in clinical trials for a number of additional indications. Other tumour antigens are currently being trialled as targets for anti-CD3 containing BiTE^®^s including BCMA, CD33, CEA, HER2, EGFR, and EpCAM [26,102].

#### 4.1.2. BiKEs & TriKEs

Bispecific killer cell engagers (BiKEs) and trispecific killer cell engagers (TriKEs) are bi- or tri- specific tandem scFvs used to redirect natural killer (NK) cells via an anti-CD16 scFv. They work in a similar fashion to BiTE^®^s. Although there are currently no BiKEs or TriKEs in clinical trials, BiKEs are currently being developed by Sanofi in collaboration with Innate Pharma using Innate Pharma’s anti-CD335 (NKp46) antibody to redirect NK cells [105].

Anti-CD16/CD33 BiKEs showed promise in an in vitro study treating myelodysplastic syndrome (MDS) [106]. Here, the anti-CD16 scFv was used to activate depleted NK cells which were redirected against myeloid-derived suppressor cells (MDSCs) expressing CD33 by the anti-CD33 scFv. The treatment was shown to reduce the immunosuppression of NK cells by MDSCs, induce MDSC cell lysis, and induce optimal MDS-NK cell function regardless of disease stage.

More recently, an anti-CD16-IL-15-anti-CD33 TriKE was also shown to overcome the cancer induced immunosuppression observed in MDS and AML [107].

#### 4.1.3. DART^®^s

Developed by MacroGenics, the DART^®^ platform has been used to produce five anti-cancer molecules, four of which entered phase I clinical trials for the treatment of AML/MDS, solid tumours, or colorectal cancer [24].

The DART^®^ platform is compatible with several modalities. Of the five anti-cancer DARTs listed, three include an anti-CD3 binding moiety to re-direct T-cells towards cells expressing the cancer antigen complimentary to the second binding site in the same fashion as a BiTE^®^. The other two DARTs, which target PD-1/LAG3 or PD-1/CTLA-4, block pathways involved in T-cell inhibition leading to an enhanced T-cell response against tumour cells [108].

Although BiTE^®^s and DART^®^s can exploit the same pathways, a 2011 study comparing a CD19/CD3 bispecific molecule in DART^®^ and BiTE^®^ formats showed increased potency of the DART^®^ compared to the BiTE^®^, which was not accompanied by an increase in non-specific T-cell activation of CD19- cell lysis in vitro [109]. The study also trialled an anti- CD19/TCR DART^®^, which showed activity in an in vivo xenograft mouse model and was virtually identical in vitro to the anti-CD19/CD3 DART^®^, describing another potential mechanism of action for the DART^®^ platform.

#### 4.1.4. ImmTAC^®^s

**Figure 11:**
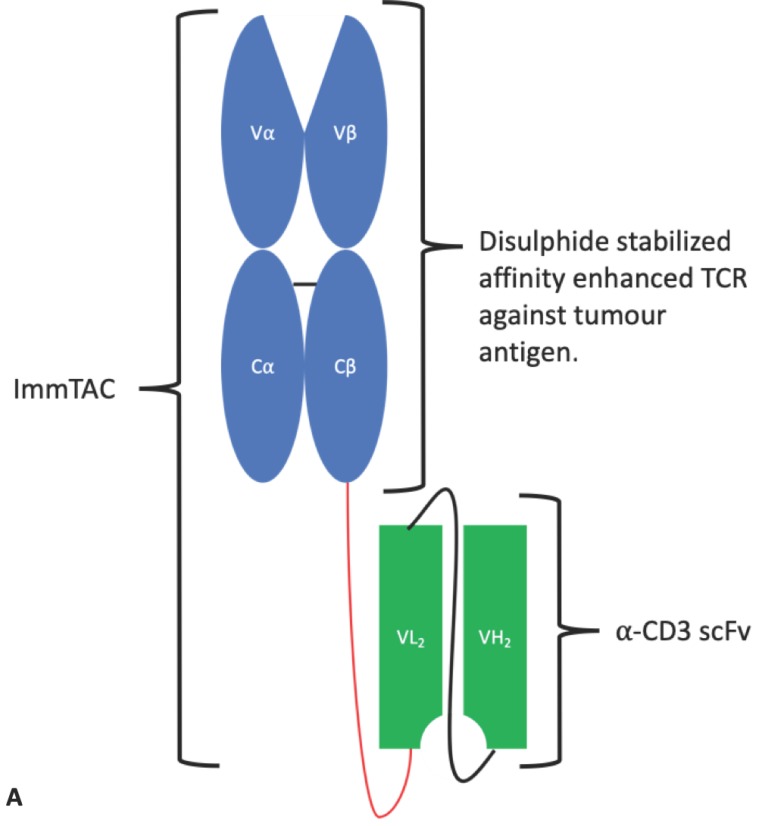

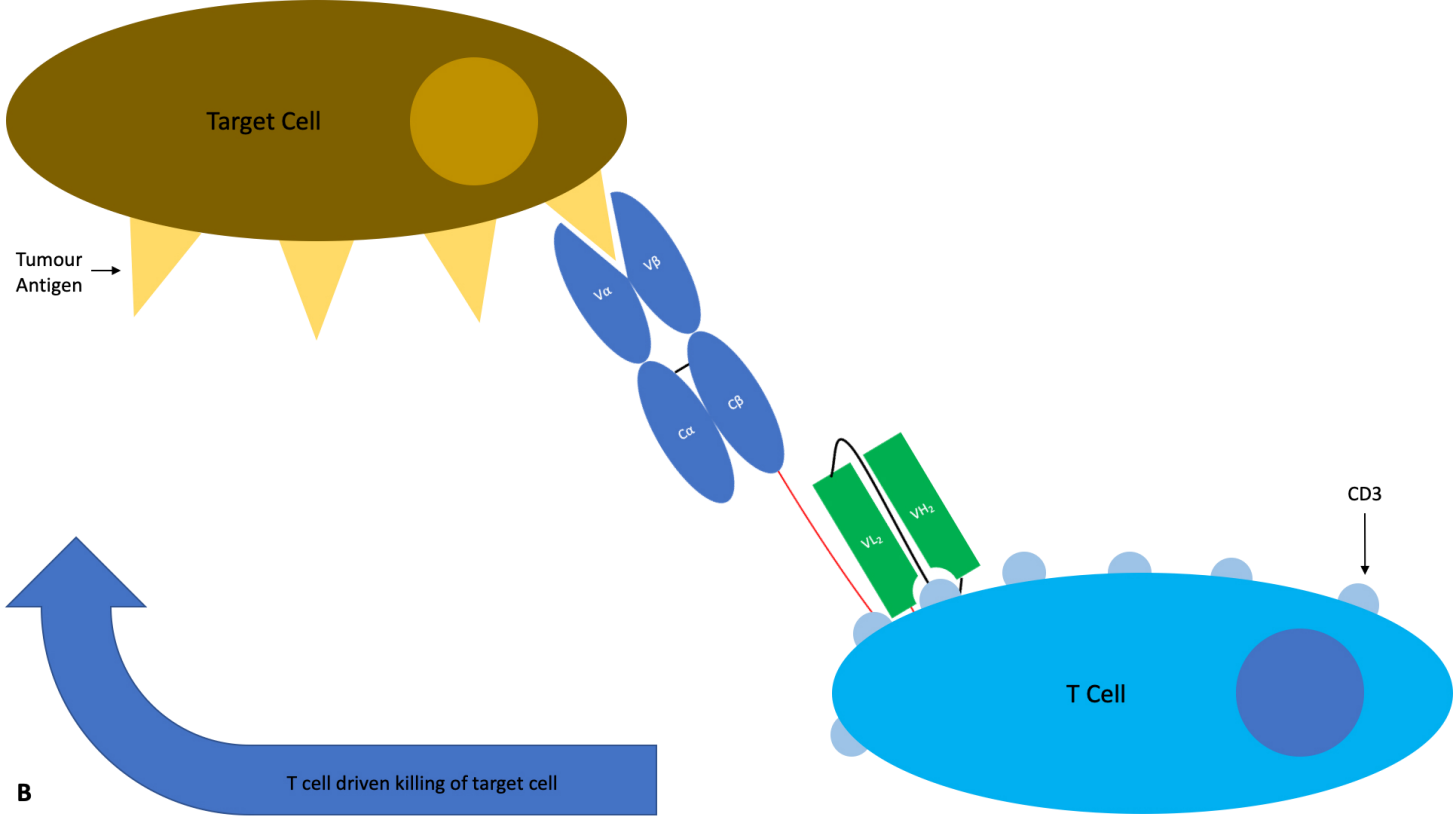
The structure and mechanism of action of ImmTAC^®^s. (**A**) The structure of an ImmTAC^®^, comprising an α-CD3 scFv linked to a disulphide-stabilised, affinity-enhanced soluble T-cell receptor. (**B**) The T-cell receptor binds its target with picomolar affinity causing the ImmTAC^®^ to cluster on the target cell. The anti-CD3 scFv then recruits passing T-cells by binding CD3 with nanomolar affinity. The clustering of CD3 on the T-cell leads to activation and re-direction of the T-cell to produce an immune response against the target cell.

The immune mobilising monoclonal T-cell receptors against cancer (ImmTAC^®^) is a novel scFv-TCR chimeric format developed by Immunocore. ImmTAC^®^s are peptide-HLA-specific, dimeric affinity-enhanced soluble TCRs containing an artificial disulphide bond, joined by a peptide linker to an anti-CD3 scFv (Figure 11). The TCR portion binds with picomolar affinity to the target cell expressing the peptide antigen in the context of MHC. The anti-CD3 portion then binds with nanomolar affinity to passing T-cells that are recruited to kill the target cell. The binding of multiple CD3 surface proteins by multiple ImmTAC^®^s on a single T-cell causes T-cell activation leading to an immune response against the target cell/tissue [110]. Importantly, ImmTAC^®^s hold the potential to overcome T-cell tolerance and the low affinity of native TCRs for cancer antigen/MHC complexes and mediate an enduring immune response [111]. An ImmTAC^®^ targeting gp100 is being tested in two phase I trials for uveal melanoma. An ImmTAC^®^ against NY ESO entered phase I clinical trials in 2018 for a variety of cancer indications, and IMC-C101C against melanoma associated antigen 4 is planned to enter phase I in 2019 [112].

As a side note, immune-mobilising monoclonal T-cell receptors against virus antigens (ImmTAV^®^s) are similar to ImmTAC^®^s in that they are composed of an affinity-enhanced soluble TCR linked to an anti-CD3 scFv. However, as the name would suggest, the TCR is designed to bind specific viral antigens as opposed to cancer antigens. These molecules are being investigated as a novel class of HIV therapy [113].

#### 4.1.5. Nanobodies^®^

Two nanobodies reached phase I clinical trials for oncology indications: ALX-0651, which targets CXCR4 for multiple myeloma and non-Hodgkin’s lymphoma, and TAS266 (Ablynx/Novartis), which targets the death receptor DR5 for solid tumours. However, their development has not been pursued [44]. On the contrary, there are a large number of nanobodies in preclinical development for a variety of other cancers. The reader is referred to the excellent review by Steeland et al. (2016) [44].

#### 4.1.6. Antibody Fragment-Drug Conjugates

In addition to PEGylation or fusion to albumin binding antibody fragments to improve half-life and pharmacokinetics, a wide range of effector moieties, including cellular toxins, radioisotopes, cytokines, and enzymes, have been conjugated to Fab, scFv, and Nb fragments. In 2010, there were 12 scFv and 12 Fab conjugates in clinical trials worldwide [4,40,114,115]. Citatuzumab bogatox, an anti-epithelial cell adhesion molecule (EpCAM) Fab conjugated to bouganin, developed by Viventia Biotechnologies Inc., is currently in phase I clinical trials for the treatment of solid tumours. Naptumomab estafenatox, an anti-trophoblast glycoprotein 5T4 (TBGP) that Fab fused to *Staphylococcus aureus* enterotoxin E, developed by Active Biotech, is currently in phase III clinical trials for renal cell carcinoma and non-small cell lung carcinoma [17].

Conjugation to cytokines is also an effective way of enhancing anti-tumour efficacy. Philogen (Switzerland) are developing a number of immunocytokines for oncology indications including Fibromun, a scFv (L19) against the tumour antigen EDB fused to TNF, Darleukin, which contains L19 scFv fused to IL-2, Teleukin, which contains a vascular targeting antibody F16 linked to IL-2, and Dodekin which contains two subunits of the immunomodulatory payload IL-12 fused to a human vascular targeting antibody in tandem diabody format [116].

Radioimmunotherapy (RIT) is the combination of radiation therapy with Ab immunotherapy and has become an attractive strategy in cancer treatment because it allows for the selective destruction of cancer cells and is less pervasive than radiotherapy. The Ab recognises and binds the surface of the primary tumour site and disseminated disease tissue and thereby delivers high doses of radiation directly to the tumour without significant damage to healthy tissue. Recent examples are the generation of radiolabelled antibodies for the radioimmunotherapy of multiple myeloma [117] and radio-iodinated anti-HER2 Nanobody^®^ for breast cancer [118].

Although the effector moieties add another mechanism through which the antibody fragments can mediate a therapeutic effect, antibody drug conjugates are not easy to develop and optimise. Many factors need to be considered, including what antibody/fragment is used, what to conjugate, what linker/chemistry to use, and the ratio of naked to conjugated antibodies [119,120].

### 4.2. Autoimmune and Inflammatory Diseases

Autoimmune diseases are chronic and potentially life-threatening, and antibody therapies are extremely expensive because they usually require intensive, life-long treatment. The lower production costs of antibody fragments and potential reduced immunogenicity due to their small size renders the use of antibody fragments with half-life extension moieties as a viable alternative to full-length antibodies. Furthermore, like for cancer immunotherapies, the development of antibody fragments for the treatment of autoimmune diseases has been growing at a fast pace and there are numerous possibilities for bispecific targeting.

One of the first antibody fragments to be marketed for an autoimmune disease indication was Certolizumab pegol (Cimzia^®^), a pegylated Fab targeting TNF developed by UCB (Belgium), approved by the FDA for the treatment of Crohn’s disease in 2008. It has subsequently been approved for rheumatoid arthritis, psoriatic arthritis, and ankylosing spondylitis. Two other Fabs are in clinical trials: FR104 (OSE/Janssen) against CD28 in phase II for RA, and Dapirolizumab, an anti-CD40L Fab developed by UCB in phase II for SLE. 

There are four nanobodies reported to be in clinical development: ALX-0061 (Vobarilizumab) is a monovalent Nanobody^®^ against IL6R linked to a half-life extending Nb against HSA from Ablynx in phase II trials for RA and SLE [121,122]. ALX-0761 (Merck/Ablynx) is a bispecific Nanobody^®^ targeting IL17A/IL17F linked to an HSA Nb in phase Ib for psoriasis [123], and Ozoralizumab or ATN-103 (Taisho) is a Nanobody^®^ against TNF. Some success has been reported for ATN-103 in a phase II interventional long-term safety study in subjects with RA at week 48 [124]. ATN-192 is a pegylated version of ATN-103, which is in phase I clinical trials [125]. Several Nbs are also reported in pre-clinical development for autoimmune disease indications [44].

One scFv format currently being evaluated in a phase II clinical study for the treatment of RA is Dekavil or F8IL10 (Philogen). It is a fully human fusion protein composed of the vascular targeting scFv antibody F8 fused to the cytokine interleukin-10. A number of other immunocytokines fused to scFvs are also in preclinical development—the reader is referred to the Philogen website for further information [126].

MacroGenics are developing MGD-010, a DART^®^ targeting CD32B and CD79B on B-cells for the treatment of autoimmune disorders. CD32B is a checkpoint molecule expressed on B lymphocytes that, when co-ligated with CD79B (a component of the B-cell antigen receptor complex), delivers a co-inhibitory signal that dampens B-cell activation. The intended mechanism of MGD010 is to modulate the function of human B-cells while avoiding their depletion. MGD010 completed phase I clinical trials in 2016 and has now been licensed to PreventionBio, who will evaluate the safety and efficacy of MGD-010, now called PRV-3279, in a phase Ib trial, expected to commence in the second half of 2019. 

Another fragment in development for a wide variety of autoimmune disease indications is ARGX-113. This is an IgG1 Fc-fragment developed by ArGenX for the treatment of patients with high levels of circulating pathogenic IgG, found in acquired thrombotic thrombocytopenic purpura (aTTP), SLE, MS, or myasthenia gravis (MG). ARGX-113 binds to the neonatal Fc receptor (FcRn) and blocks IgG recycling, resulting in clearance of autoreactive antibodies through lysosomal degradation. ARGX-113 showed statistically significant improvement in a phase II clinical trial on patients with MG [127] and is currently being evaluated for efficacy, safety, and tolerability in a randomised, double-blind, placebo-controlled, multicentre phase III trial in patients with MG having generalised muscle weakness [128].

The last category of antibody fragments tested in clinical trials for autoimmune and inflammatory diseases are dAbs. Lulizumab pegol, a pegylated Domain Antibody^®^ targeting CD28 developed by Bristol-Myers Squibb, was evaluated in a phase II trial in subjects with active systemic lupus erythematosus (SLE). There was no significant difference between lulizumab and placebo for the primary (BICLA response rate) or secondary endpoints at week 24, although PD activity was observed [129]. 

GSK has developed two inhalable anti-TNFR1 VH domain antibodies for selective antagonism of TNF in the lung interstitium for acute respiratory distress syndrome (ARDS)/acute lung inflammation (ALI). Delivery of antibody fragments directly into the lung by inhalation has great potential for treatment of inflammatory lung diseases, the advantages being rapid onset of action, reduced systemic exposure, lower doses, as well as needle-less administration. GSK1995057 was tested in a phase I clinical trial in healthy volunteers. It was not developed further because of the presence of naturally occurring, pre-existing anti-drug antibodies (ADAs) which could lead to early neutralising anti-drug-antibody responses [130]. GSK1995057 was subsequently engineered by adding a C-terminal alanine residue to render it less susceptible to ADAs. The resultant GSK2862277 was tested in a phase I trial and was well tolerated when administered both as an orally inhaled aerosol and by iv route. A phase II placebo-controlled randomised trial in patients that were undergoing esophagectomy surgery and were at risk to develop ARDS has been completed [131] with results expected in 2019. 

### 4.3. Other Clinical Applications

While oncology and autoimmune disease are two major areas in which antibody fragments have become a prominent class of therapeutic molecules, there are several other disease areas in which, although not as dominant, fragments are being evaluated.

#### 4.3.1. Ophthalmic Indications

Antibody fragments such as Fabs and scFvs, unlike full-length antibodies, have been shown to be able to penetrate the cornea and pass into the eye and achieve clinically useful concentrations in the anterior chamber over a reasonable time-span following topical administration [132] but to date there are no reports of this route of administration being tested in the clinic. Most are administered by direct injection into the eye (intravitreal route).

The most common eye disorder treated with antibodies or antibody fragments is age-related macular degeneration (AMD), which is the leading cause of irreversible blindness in people aged 50 years or older, in the developed world. For AMD, the antibody fragments are applied directly to the eye via the intravitreal route. Extremely high local drug concentrations can be achieved in the eye with minimal risk of systemic side effects.

Ranibizumab (Lucentis^®^) is an anti-angiogenic monoclonal antibody fragment targeting VEGF-A, derived from the same parental mouse antibody as bevacizumab. It was approved in 2006 for wet AMD and subsequently in 2012 and 2015 for diabetic macular oedema and diabetic retinopathy, respectively. 

Lampalizumab (Roche), a Fab against complement factor D, entered phase III clinical trials for geographic atrophy, an advanced form of age-related macular degeneration [133,134] in 2014 but failed to meet primary endpoints [135].

Brolucizumab (Alcon/Novartis) is a scFv targeting VEGF that is currently in phase III for wet AMD [136,137].

A number of antibody fragments are also in preclinical development for eye indications [138,139]. Elasmogen is developing V-NARs such as ELN/21, an ICOSL G-binding soloMER^TM^, in preclinical development for posterior uveitis and corneal graft rejection, and ELN/12, an anti-VEGF soloMER^TM^ for AMD [140]. Abzyme has a bivalent nanobody targeting VEGF and TfR for wet AMD.

#### 4.3.2. Infectious Diseases

Only three full-length mAbs have been approved for the treatment of infectious diseases: Synagis^®^ (Palivizumab) for RSV infection, Abthrax^®^ (Raxibacumab) against anthrax, and Zinplava^TM^ (Bezlotoxumab) against *C. difficile* (although technically the latter two mAbs neutralise bacterial toxins—see below) and there are currently over 60 mAbs in various stages of clinical trials for the treatment of infectious diseases including Ebola, hepatitis B, and respiratory syncytial virus (RSV). The development of antibody fragments in infectious diseases is under-exploited, likely due to their lack of effector function. To our knowledge, there are only three molecules currently in clinical trials and no approved therapies. Afelimomab is an F(ab’)_2_ in phase III trials for sepsis toxic shock [17]. Rivabazumab pegol is a pegylated Fab in phase II for the treatment of chronic *Pseudomonas aeruginosa* infection [17]. The third is ALX-0171, a trivalent Nanobody^®^ (VHH_3_) in phase III for the treatment of RSV infection [17]. ALX-0171 binds to RSV F protein. The potency of the trivalent ALX-0171 against RSV-A and RSV-B strains was found to be several thousand-fold higher than that of the monovalent nanobody. It is also the first Nanobody^®^ treatment developed for delivery directly into the lungs, the site of RSV infection, by nebulisation [141]. Unfortunately, Sanofi decided to stop development ALX-0171 in Feb 2019 [142].

Although ALX-0171 remains the only Nb to reach clinical trials in this therapy area, there have been many in vivo and in vitro studies investigating the use of nanobodies against a wide range of bacteria, viruses, parasites, and fungi, including rotavirus, norovirus, HIV-1, *Helicobacter pylori*, *Trypanosoma brucei*, and *Plasmodium falciparum.* The reader is directed to two recent reviews by Steeland et al. and Wilken and McPherson [44,141].

#### 4.3.3. Anti-Toxins and Anti-Venoms

Traditionally, the treatment for envenoming has been the transfusion of serum from immunised animals. Primarily containing IgG, Fab and F(ab’)_2_ fragments, much of which will not be specific for the venom, serotherapy can have several undesirable affects including IgE-mediated and non-IgE-mediated early adverse reactions, anaphylaxis and serum sickness [143]. The ability of antibody fragments to rapidly penetrate tissue, their lack of effector function, and their retained specificity and affinity for their antigen makes them promising candidates for anti-venoms. In addition, they can easily be produced recombinantly in a homogeneous form. 

Although none have progressed to the clinic as yet, many Nbs have been generated that have shown the ability to neutralise toxins/venoms in in vitro and in vivo models. Venoms from *Androctonus australis* (Fat tailed scorpion), *Hemiscorpius lepturus* (Iranian scorpion), and *Naja kaouthia* (monocled cobra) and toxins from *Clostridium difficile*, *Vibrio cholera*, *Bacillus anthracis*, and *E. coli*, including Shiga toxins 1 and 2, have all been neutralised [44,144].

## 5. Non-Therapeutic Uses

### Imaging & Diagnostics

The use of antibodies for molecular imaging is well established. In essence, their high affinity and specificity make them ideal for the detection of a specific surface protein in vivo or in vitro. Additionally, their large size allows for their conjugation to radioisotopes, fluorescent molecules, or even enzymes without inhibiting binding to their target [145].

However, conventional antibodies are by no means perfect for in vivo imaging. Their long half-lives and low rates of clearance necessitates a several-day waiting period to obtain an acceptable signal-to-noise ratio, exposing patients to excessive radiation from radioisotopes. In addition, potential off-target immune effects, conferred by the Fc domain, are undesirable. Antibody fragments, either produced recombinantly or by enzymatic cleavage, provide a solution to these downsides. Fragments lack an Fc domain, thus removing their immune activating potential and reducing their half-life simultaneously. This reduces the risk of disturbing the system being visualised, allows for rapid high-contrast imaging, and reduces radiation exposure [145]. In addition, their small size allows for better tissue distribution and provides more options with regard to epitope choice [12]. Nanobodies targeting CAIX and HER2 have been used for optical imaging of pre-invasive breast cancer, which requires a high tumour to background ratio [146,147].

Diabodies, tribodies, and tetrabodies also have potential uses in applications such as radioimmunotherapy and diagnostic in vivo imaging [148,149]. In addition, fluorescently labelled nanobodies have been used for real-time analysis of epithelial mesenchymal transition [150].

The applications of single-domain antibodies in in vivo imaging and diagnostics are not restricted to oncology. In 2014, an anti-VCAM1 single-domain antibody fragment was shown to be an accurate and reproducible tool for the imaging of atherosclerotic lesions [151,152].

## 6. Future Opportunities

### 6.1. Neurodegenerative Diseases

Antibody therapies have traditionally been thought to be of limited relevance in the treatment of neurodegenerative disease due to the miniscule proportion of antibodies in circulation that can cross the blood–brain barrier (BBB) [153]. The four monoclonal antibodies approved for the treatment of multiple sclerosis—natalizumab (Tysabri^®^), alemtuzumab (Lemtrada^®^), daclizumab (Zinbryta^®^), and ocrelizumab (Ocrevus^®^)—are thought to mediate their effects primarily in the periphery [154]. There are also two antibodies against alpha synuclein (PRX002/RO7046015 from Roche and BIIB-054 from Biogen) entering clinical trials for Parkinson’s disease. No antibody fragments have yet progressed into the clinic.

The causal mechanism of many neurodegenerative diseases, including Alzheimer’s and Huntington’s disease, involves aggregation of misfolded protein [155], and it has been shown that it is possible to raise antibodies that can neutralise these toxic aggregates. One possible approach to circumvent the BBB challenge is to express antibody fragments, termed intrabodies, within the cells of the brain. Although this approach may be far from reaching clinical trials, partially due to its invasive nature, it has been shown to be efficacious in some in vivo models [156]. Penetration of the BBB is discussed in Section 6.2.

### 6.2. Cell and Tissue Specific Antibody Delivery

Bispecific antibodies where one specificity is used to target the antibody to a specific tissue or cellular compartment and the second specificity is used to target the antigen of interest would have great advantages in limiting off-target effects due to systemic administration. Conditions in which the target of interest is located in the central nervous system (CNS) are particularly challenging, however, as most antibodies are generally unable to penetrate the BBB. Receptor-mediated transcytosis (RMT) is an example of a macromolecule transport system that is employed by cells of the BBB to supply essential proteins to the brain. This system can be utilised to deliver biologic payloads, such as antibodies, across the BBB. Increased brain penetration of therapeutic antibodies can be achieved by engineering bispecific antibodies in which one antibody binding specificity recognises a BBB receptor that undergoes RMT from the circulatory compartment into brain parenchyma, and the second binding specificity recognises a therapeutic target within the CNS. Anti-transferrin receptor (TfR)-based bispecific antibodies have previously shown promise for boosting antibody uptake in the brain [157]. Abzyme Therapeutics are now exploiting modular anti-TfR antibodies (nanobodies) and TFR-directed bispecifics capable of overcoming the blood–brain barrier for treatment of CNS disorders [158].

Some fragment formats, including F(ab’)_2_ and basic VHHs, have been demonstrated to cross the BBB with relative efficiency, although they may not necessarily accumulate to therapeutic concentration due to rapid clearance [159,160,161]. Engineering these formats to bind key proteins in the causal mechanism of neurodegenerative diseases will likely be challenging, but their existence shows the significant progress that has been made in recent years. In 2001, Muruganandam et al. identified two single-domain antibody fragments of camelid origin capable of crossing the BBB endothelium, FC5 and FC44, by phenotypic panning of a naive llama single-domain antibody phage display library [162]. Recently, Farrington et al. engineered FC5 as a mono- and bivalent fusion with the human Fc domain and showed up to a 30-fold enhanced apparent brain exposure (derived from serum and cerebrospinal fluid pharmacokinetic profiles) compared with control domain antibody-Fc fusions after systemic dosing in rats [163]. This study demonstrates that modular incorporation of FC5 as the BBB carrier arm in bispecific antibodies or antibody-drug conjugates may have potential use in the development of pharmacologically active biotherapeutics for CNS indications. As an alternative, Caljon et al. suggested grafting CDR loops of BBB penetrating Nbs onto an as-yet undiscovered scaffold to combine BBB penetration and high antigen specificity with the desired pharmacokinetic properties [161]. 

### 6.3. Intracellular Targeting

Antibodies have proven to be effective at modulating a wide variety of disease associated molecules belonging to different target classes, but there are still hundreds of disease-associated intracellular targets that are inaccessible to antibodies and undruggable with small molecules and that include phosphatases, E3 ubiquitin ligases, GTPases, and transcription factors. The majority of antibodies are unable to penetrate into cells. Thus, while small molecules drugs can easily penetrate cell membranes to hit intracellular targets, they often lack specificity, in particular when multiple targets have similar binding pockets. Furthermore, their small size makes them ineffective at blocking certain protein–protein interactions where large interfaces are involved. Antibodies or antibody fragments can solve the specificity problem and effectively block protein–protein interactions, but they have been largely restricted to targets in the extracellular milieu because they cannot cross the lipid bilayer. It has therefore been the ‘holy grail’ of many pharmaceutical companies to combine the targeting power of monoclonal antibodies with the cell-penetrating abilities of small molecules. Even if cell penetration can be achieved, a further complication is the need for tissue and cellular specificity to limit off-target effects, although one could argue that cell-specific action can be reached on the intracellular antigen level. 

A number of different approaches have been described in the literature including transfection, cell penetrating peptides (CPPs), fragments of bacterial toxins, nanocarriers (lipid-based, polymer-based, and virus- and virus-like particle-based), and physical methods such as microinjection and electroporation and have recently been extensively reviewed [164]. Below we briefly describe some of the most promising approaches that could be applicable for the systemic delivery of antibody fragments.

A cell-penetrating, intracellular targeting antibody needs to bind to a receptor on the cell surface and trigger internalization, for example, by receptor-mediated endocytosis. Once internalised, the antibody needs to be able to escape from the endosome in order to bind to/neutralise its intracellular target in the cytosol, where the majority of potential therapeutic targets are concentrated, or in the nucleus. 

A frequently used approach for intracellular delivery is the fusion of an antibody or antibody fragments including scFv, Fab, and nanobody to a CPP. Early studies showed that HIV tat and other CPPs such as the membrane translocating sequence from Kaposi fibroblast growth factor, the Antennapedia protein transduction domain, the Penetration of the Drosophila homeodomain, nona-arginine, and certain oligonucleotides could cross the plasma membrane to enter cells. However, their mechanism of intracellular delivery was unclear, and it is unknown if/how CPPs and CPP-conjugated antibody fragments are released from endocytotic vesicles, making efficient endosomal escape one of the limitations of this approach. Other limitations include the lack of optimisation for intracellular localization and the lack of cell or tissue-specific targeting. The latter may be solved by addition of a cell-targeting ligand to the antibody fragment. This was elegantly demonstrated by the authors in [165], who fused a cancer-cell specific, 17-amino acid peptide (BR2) to anti-mutated K-ras scFv, and demonstrated significant and cancer-cell-selective effects in vitro. 

Most currently used CPPs seem to contain a disproportionately high number of positively charged lysine and/or arginine residues and have a high theoretical net charge. These positively charged residues facilitate interaction with negatively charged cell surface proteoglycans, such as sulfated proteoglycans like HSPG, ultimately enabling cellular uptake. These findings suggested that polycationic protein resurfacing could endow cell penetration properties, the downside being that extensive mutation of most proteins would lead to a loss of functional activity. To this end, Bruce et al. recently showed that nanobodies are amenable to cationic resurfacing [166]. Structural analysis of a GFP-binding nanobody revealed a number of solvent exposed residues that were not within the CDRs. Polycationic resurfacing of these solvent exposed residues resulted in a new protein that expressed well in *E. coli*, retained affinity for GFP, and penetrates mammalian cells. Analogous mutation of HER2-or β-lactamase-binding nanobodies also resulted in well-expressed nanobodies that exhibited potent cell-penetrating properties, and the majority of the internalised proteins were found to reside in the cytosol, although the mechanism of uptake remains unclear. 

Probably one of the most exciting recent technology developments in the field of intracellular targeting has come from Orum Therapeutics (South Korea). Orum have developed a cell-penetrating antibody technology that uses a cell surface receptor-specific cyclic peptide fused to an antibody targeting activated K-ras that has been engineered from a naturally occurring autoimmune antibody able to penetrate into cells. The peptide, which confers cell-type specificity, is genetically linked to the light chain variable domain of the antibody. The light chain contains a sequence motif in L-CDR3, which enables the antibody to escape from acidified endosomes into the cytosol, where the heavy chain of the antibody is able to engage with and neutralise its intracellular target, activated K-ras [167]. Using this strategy, a humanised IgG1 format antibody named iMab RT11-i fused to a tumour-homing αv integrin binding RGD10 cyclic peptide was developed, and this had significant anti-tumour effects in vivo in a tumour xenograft model in mice, demonstrating the feasibility of this approach for the cytosolic delivery of antibodies. Based on this approach, it is conceivable that antibody fragments, e.g., nanobodies or domain antibodies with different targeting specificities, could be combined to achieve a similar effect. 

## 7. Conclusions

In this review we have covered the structure of a range of antibody fragments, from the isolated domains of canonical IgG to nanobodies, their production in microbial prokaryotic and eukaryotic, cell-free systems, and their application in and outside of the clinic. We have discussed the main advantages of each fragment format with a focus on size and effector function and have highlighted the main mechanisms of action through which these fragments mediate their therapeutic effects.

Since the approval of muromonab in 1986, antibodies have rapidly expanded to become a major class of therapeutic molecule and are essential to the way we treat many diseases. Therapies have also diversified from the canonical mAb to a wide range of fragments and antibody-drug conjugate formats, which can offer context-dependent improvements to full-sized mAbs, the most common of which are discussed here. There is nothing to suggest that this trend will not continue with new formats and artificial frameworks (not discussed in this review) constantly being developed and gradually making their way into the clinic.

New formats offer exciting opportunities to expand the uses of antibodies into previously uncharted territory. For example, an orally taken antibody for the treatment of neurological diseases or antibodies against intracellular targets were once thought impossible due to an antibody’s large size and susceptibility to acidic proteases. Small, highly stable, even at low pH, nanobodies that are easy to link in series may potentially offer a way to access previously undruggable targets and tissues whilst being completely un-invasive. Fragments allow us to break central tolerance and re-target a host’s immune cells against cells displaying previously unrecognised cancer antigens in tissues too far removed from circulation for conventional antibodies to access. Fragments allow us to discover new antibodies at an astounding rate, create a vast array of multi-specific molecules, and rapidly search for indicators of disease almost anywhere in the body. 

Although still not without their limitations and complications, the future of antibody-derived fragments undoubtedly looks bright. The diverse range of formats and modifications available combined with yet to be explored sequence space may allow us to overcome the challenges that we face in this modern era, from diseases of the wealthy and old aged, to the infectious and transmissible. 

## Abbreviations

6HIS	hexa-histidine
ADA	anti-drug antibody
ADCC	antibody dependant cellular cytotoxicity
ADCP	antibody dependant cellular phagocytosis
ADH1	alcohol dehydrogenase 1
AIEX	anion exchange chromatography
ALI	acute lung inflammation
ARDS	acute respiratory distress syndrome
aTTP	acquired thrombotic thrombocytopenic purpura
ALL	acute lymphoblastic leukaemia
AMD	age-related macular degeneration
AML	acute myeloid leukaemia
BBB	blood–brain barrier
BCMA	B-cell maturation antigen
BiKE	bispecific natural killer cell engager
BiTE^®^	bispecific T-cell engager
CDC	complement-dependent cytotoxicity
CDR	complementarity determining region
CEA	carcinoembryonic antigen
CHO	Chinese hamster ovary
CIEX	cation exchange chromatography
CL	constant domain of immunoglobulin light chain
CNS	central nervous system
CPP	cell penetrating peptide
dAb	Domain antibody^®^
DART^®^	dual affinity re-targeting protein
EpCAM	epithelial cell adhesion molecule
ER	endoplasmic reticulum
Fab	fragment of antigen binding
Fc	fragment crystallizable
FcRn	neonatal fragment crystallizable receptor
Fv	fragment variable
GAPDH	glyceraldehyde-3-phosphate dehydrogenase
GFP	green fluorescent protein
GM-CSF	granulocyte-monocyte colony stimulating factor
GST	glutathione-S transferase
HAVH	human anti-VH
HEK	human embryonic kidney
HER2	human epidermal growth factor 2
HIC	hydrophobic interaction chromatography
HIV	human immunodeficiency virus
HLA	human leukocyte antigen
IgG	immunoglobulin gamma
IMAC	immobilised metal affinity chromatography
ImmTAC^®^	immune mobilising monoclonal t-cell receptors against cancer
ImmTAV^®^	immune mobilising monoclonal t-cell receptors against virus antigens
mAb	monoclonal antibody
MBP	mannose binding protein
MDS	myelodysplastic syndrome
MDSC	myeloid derived suppressor cell
MG	myasthenia gravis
MHC	major histocompatibility complex
MS	multiple sclerosis
Nb	Nanobody^®^
NK cell	Natural killer cell
NY ESO	New York esophageal squamous cell carcinoma
PD	pharmacodynamic
PEG	polyethylene glycol
PGK1	phosphoglycerate kinase 1
RMT	receptor-mediated endocytosis
RSV	respiratory syncytial virus
scFv	single chain fragment variable
SEC	size exclusion chromatography
SLE	systemic lupus erythematosus
TBGP	anti-trophoblast glycoprotein 5T4
TCR	T-cell receptor
TfR	transferrin receptor
TNF	tumour necrosis factor
IL	interleukin
TNFR1	tumour necrosis factor receptor 1
TriKE	trispecific natural killer cell engager
VEGF	vascular endothelial growth factor
VH	variable domain of immunoglobulin heavy chain
VL	variable domain of immunoglobulin light chain
V-NAR	variable new antigen receptor
vWF	von Willebrand factor
